# DNA microarray gene expression profile of bovine macrophages cell line (BoMac) after infection with Bovine Immunodeficiency Virus or Bovine Foamy Virus

**DOI:** 10.1186/1742-4690-8-S1-A22

**Published:** 2011-06-06

**Authors:** Marzena Rola, Magdalena Materniak, Aneta Pluta, Jacek Kuzmak

**Affiliations:** 1 Department of Biochemistry, National Veterinary Research Institute, Pulawy, 24-100, Poland

## 

Bovine Immunodeficiency Virus (BIV) and Bovine Foamy Virus (BFV) are frequently found in cattle under natural conditions. However, the effect of their infection on immune functions has not been fully characterized. To investigate that, genes expression in BoMac cells was determined after in vitro infection with BIV or BFV. BLOPlus microarrays (Michigan State University) were applied for transcriptome analysis. The experiment based on total RNA isolated from BoMac cells infected with BIV or BFV and from mock-inoculated cells used as a control. Labeled cDNA samples from BIV or BFV infected BoMacs were hybridized independently against controls in four repeats. Differentially expressed genes were defined as those that had absolute fold change ratios greater than 1.5 ( p≤0.1). 173 and 122 genes have shown changes in expression level after BIV and BFV infection, respectively. The DAVID software was used to asses the enrichment of annotation terms of analyzed genes. Ontological analysis based on KEGG pathway database resulted in 10 terms which included: pathways in cancer, MAPK signaling pathway, cell cycle, PPAR signaling pathway and ubiquitin mediated proteolysis. The BFV gene list resulted in 6 terms that were considered significantly enriched and included those associated with: pathways in cancer, adherens junction, TGF-beta signaling pathway and endocytosis. Further analysis of respective genes with altered expression following BIV or BFV infection is required.

